# Bronchodilator Reversibility of Spirometry and Oscillometry Ratios in Severe Refractory Asthma

**DOI:** 10.1111/cea.70135

**Published:** 2025-08-10

**Authors:** Robert Greig, Philipp Suter, Rory Chan, Brian Lipworth

**Affiliations:** ^1^ Scottish Centre for Respiratory Research Ninewells Hospital and Medical School Dundee Scotland UK

**Keywords:** asthma, bronchodilator, oscillometry ratio, reversibility, spirometry ratio

**Summary**

- Oscillometry derived ratios show significant improvement to short acting beta agonists.
- Oscillometry derived ratios and spirometry ratios are significantly correlated.

To the Editor,

Bronchodilator response (BDR) is a key tenet in the diagnosis of asthma and is defined as an improvement in the forced expiratory volume in 1 s (FEV_1_) of 12% and 200 mL in response to a short acting beta agonist (SABA) such as salbutamol [1]. Oscillometry demonstrates a greater sensitivity to detect BDR when compared to spirometry in more severe asthma patients [2]. Airwave oscillometry (AOS) is an effort independent technique where a vibrating mesh creates soundwaves that are superimposed on normal tidal breathing. This allows measurement of respiratory impedance comprising components of resistance (R) and reactance (X) calculated from the relationship of pressure/flow (kPa/L/s) to frequency (Hz). AOS also facilitates the assessment of the small airways through the heterogeneity in lung resistance between total airways at 5 Hz (R5) and the large airways at 20 Hz (R20) as R5‐R20 or as peripheral lung compliance through the area under the reactance curve (AX) between 5 Hz and the resonant frequency (Fres) where the impedance equals zero. Spirometry, in comparison, measures the forced expiratory dynamic lung volumes and flow using effort dependent measurements. Oscillometry derived ratios akin to the FEV1/FVC ratio have been proposed as a method to aid the interpretation of the raw values which clinicians may not be familiar with. An R5‐R20/R5 ratio ≥ 19% or an X5/AX ratio < 15% is associated with poorer symptom control and increased exacerbations [3], and such ratios have been shown to identify significant improvements in asthma patients who were commenced on tezepelumab [4]. Here we review bronchodilator effects on both spirometry and oscillometry derived ratios.

The screening data from two previous prospective clinical trials (EudraCT No. 2019‐003763‐22 and EudraCT 2021‐005593‐25) was reviewed in patients with an abnormal R5‐R20/R5 ratio ≥ 19% who were initially identified. Prior to their pulmonary function tests, medications were withheld as follows: inhaled corticosteroid for 12 h, long‐acting beta‐agonists and muscarinic antagonists for 12 h/24 h depending on dosing frequency, SABA for 6 h, and montelukast for 48 h. None of the patients were taking biologics at the time of BDR assessment. Following the European Respiratory Society guidelines and manufacturer's instructions, fractional exhaled nitric oxide (FeNO) was obtained using NIOX Vero (NIOX, Oxford, UK) [5]. Spirometry (Micromedical, Chatham, UK) and AOS (Thorasys Tremoflo, Montreal, Canada) were performed in triplicate [6]. Using a pressurised metered dose inhaler with an Aerochamber spacer device (Trudell Medical, London, Canada) 400 μg of salbutamol was administered to all patients, with oscillometry and spirometry repeated after 20 min. SPSS version 29 was used for statistical analysis. Paired Student's *t*‐tests were applied with a two‐tailed alpha error of 5%. Nominal *p* values are quoted as either *p* < 0.05, < 0.01, or < 0.001 (two tailed). Correlations were assessed using Pearson's test.

Forty‐one patients were identified with an R5‐R20/R5 ≥ 19% with 24 male and 17 female. The mean (SEM) baseline demographics were: age 55.29 years (2.14), BMI 30.90 kg/m^2^ (4.49), beclometasone dipropionate inhaled corticosteroid dose equivalent 1415 ug (69) with 20 patients at GINA step 4 and 21 patients at GINA step 5, eosinophils 522 cells/uL (45), FeNO 57.6 ppb (7.7), asthma control questionnaire 3.09 (0.16).

The mean (SEM) baseline spirometry and oscillometry ratios were: FEV_1_/FVC 60.77% (1.93), FEF_25‐75_/FVC 34.33% (2.73), R5‐R20/R5 34.01% (1.58), X5/AX 10.75% (0.50).

The mean (95% CI) absolute % changes in the ratios in response to salbutamol were: FEV_1_/FVC 2.52% (1.23, 3.82) *p* < 0.001, FEF_25‐75_/FVC 3.94% (1.29, 6.60) *p* < 0.01, R5‐R20/R5 8.23% (4.94, 11.49) *p* < 0.001, X5/AX 5.36% (2.56, 8.17) *p* < 0.001.

Correlations between the spirometry and oscillometry ratios are shown in Table 1. There were no correlations between the change in spirometry or oscillometry ratios and either baseline eosinophils or FeNO.

**TABLE 1:** Correlation between absolute % change in spirometry ratios and oscillometry ratios in response to salbutamol for patients with a R5‐R20/R5 ratio > 19% at baseline.

	FEV_1_/FVC		FEF_25‐75_/FVC	
	*R*	*p*	*R*	*p*
R5‐R20/R5	−0.47	< 0.01	−0.54	< 0.001
X5/AX	0.50	< 0.001	0.65	< 0.001

Our data shows both oscillometry derived ratios show a significant correlation with both FEV_1_/FVC and FEF_25‐75_/FVC, with the stronger correlation being to FEF_25‐75_/FVC. This is as one might expect, given FEF_25‐75_ has been shown to be a reliable marker of small airways dysfunction [7].

We appreciate our study is a single centre study with a relatively limited sample size. As such, further studies with an increased cohort size would be indicated to validate our findings and provide further information on the oscillometry derived ratios in relation to BDR. Further research to compare the oscillometry ratios with *Z*‐scores would also be beneficial given the recently proposed *Z*‐scores for the absolute values to assess significant BDR [8].

In conclusion, both spirometry and oscillometry derived ratios show significant improvements to bronchodilation with significant correlation.

## Author Contributions

**Robert Greig:** statistical analysis and writing. **Philipp Suter:** statistical analysis and writing. **Rory Chan:** trial design and submission, review. **Brian Lipworth:** trial design, data interpretation and analysis, writing.

## Ethics Statement

07/02/2022, 21/WS/0151, West of Scotland REC 1, EudraCT2021‐005593‐25 and 09/12/2019, 19/ES/0134 East of Scotland REC, EudraCT 2021–005593‐25.

## Conflicts of Interest

Dr. Greig reports personal fees (talks) from AstraZeneca. Dr. Suter reports personal fees (talks) from AstraZeneca, personal fees (talks) from GSK, grants from Lung League Fribourg (Switzerland). Dr. Chan reports personal fees (talks) and support attending ERS from AstraZeneca, personal fees (consulting) from Vitalograph, and personal fees (talks) from Thorasys. Dr. Lipworth reports non‐financial support (equipment) from GSK; grants, personal fees (consulting, talks and advisory board), other support (attending ATS and ERS) and from AstraZeneca; personal fees (talks and consulting) from Sanofi, personal fees (consulting, talks and advisory board) from Circassia in relation to the submitted work; grants, personal fees (consulting, talks, advisory board), other support (attending ERS) from Teva, personal fees (talks and consulting), grants, and other support (attending ERS and BTS) from Chiesi, personal fees (consulting) from Lupin, personal fees (consulting) from Glenmark, personal fees (consulting) from Dr. Reddy, personal fees (consulting) from Sandoz; grants, personal fees (consulting, talks, advisory board), other support (attending BTS) from Boehringer Ingelheim, grants and personal fees (advisory board and talks) from Mylan outside of the submitted work; and the son of BJL is presently an employee of AstraZeneca.

## Data Availability

The data that support the findings of this study are available from the corresponding author upon reasonable request.
